# Meconium periorchitis presenting as a para-testicular mass outside of the tunica vaginalis: case report

**DOI:** 10.1093/jscr/rjag385

**Published:** 2026-09-01

**Authors:** Ash N Zawerton, Robert E Steckler

**Affiliations:** Geisinger College of Health Sciences, Scranton, PA, United States; Geisinger College of Health Sciences, Scranton, PA, United States

**Keywords:** meconium, periorchitis, para-testicular mass, tunica vaginalis

## Abstract

Meconium periorchitis is an uncommon but well-documented phenomenon by which intra-abdominal meconium reaches the scrotum via a patent processus vaginalis. This classically presents in a child first as a soft hydrocele, and later as a firm scrotal mass once calcification of the meconium occurs. We report a case of a 6-month-old boy with exam findings suspicious for perinatal testicular torsion who was taken for surgical exploration and found to have a meconium fecalith located inferior to the testicle and outside of the tunica vaginalis within the gubernaculum. The fecalith was excised and orchidopexy was performed. Meconium periorchitis needs to be considered in the differential diagnosis of pediatric scrotal masses. Overlooking this often-benign condition may lead to unnecessary surgery, potentially resulting in compromise of the testicle due to deliberate removal or unintended iatrogenic injury.

## Introduction

Meconium periorchitis results from an inflammatory reaction when intra-abdominal meconium reaches the scrotum via a patent processus vaginalis. Spillage of meconium from the fetal gastrointestinal tract is often associated with more severe underlying pathology, such as intestinal obstruction, volvulus, cystic fibrosis, and vascular compromise, among others [1]. Clinically, patients classically present with a painless palpable testicular mass in the perinatal period. Radiographically, the mass is classically described with the triad of a calcified scrotal mass with an associated hydrocele and no doppler flow on ultrasound [2]. Given the pathophysiology, there are often concomitant abdominal calcifications which may help confirm the diagnosis [3]. If there is a high degree of clinical suspicion, surgical intervention is not necessary, as the clinical course is typically benign and self-resolving with no known long-term impact on testicular function [1].

## Case report

**Figure 1:**
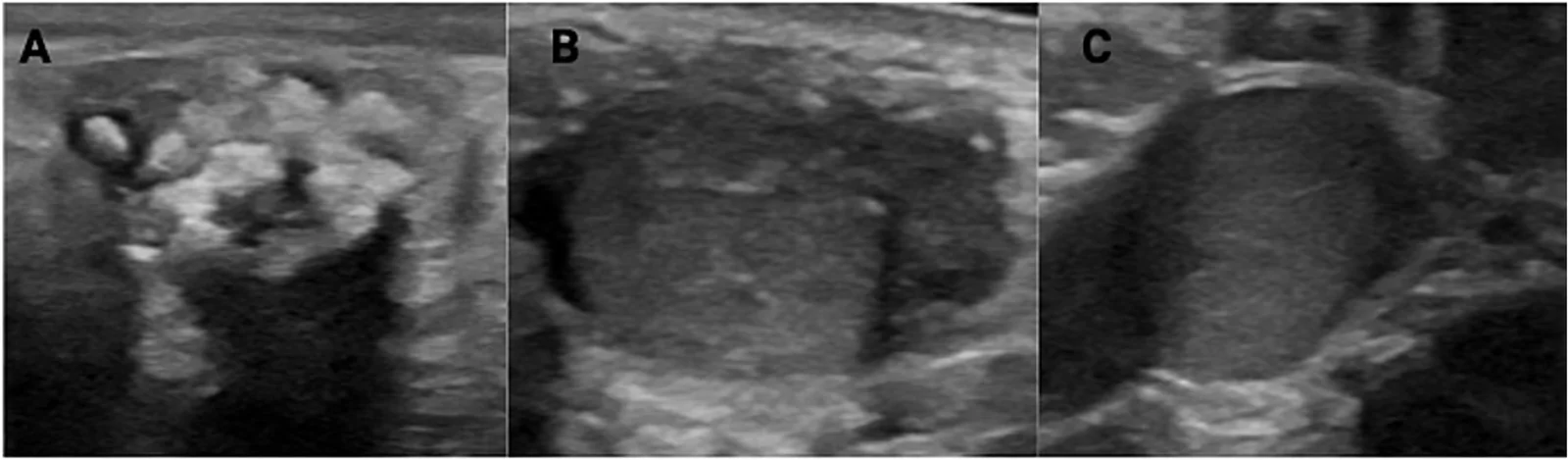
Ultrasound images demonstrating (A) meconium fecalith and (B, C) an otherwise normal appearing right testicle.

**Figure 2:**
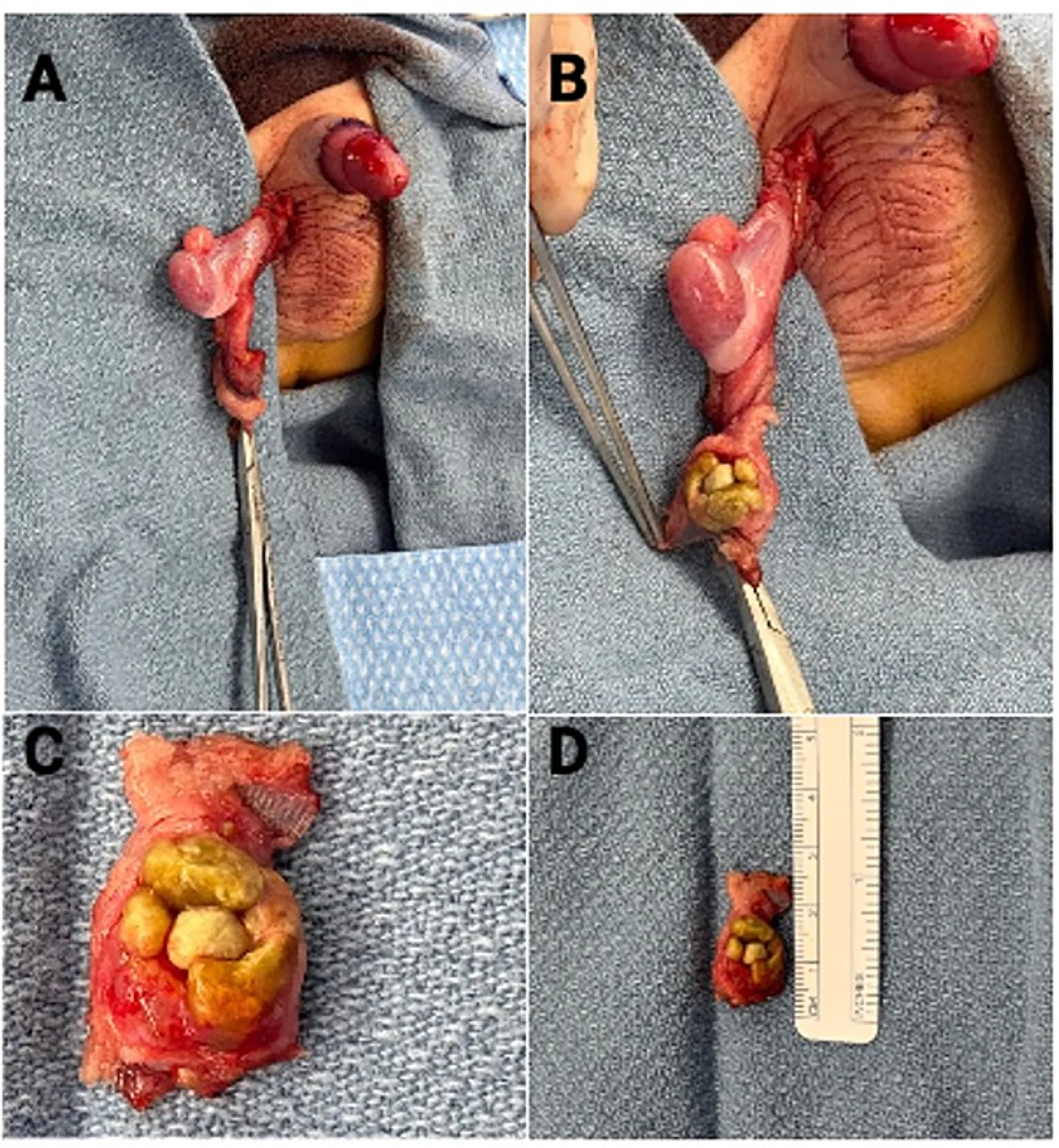
Appearance of meconium fecalith in situ (A, B) and after excision (C, D) located inferior to the testicle and external to the tunica vaginalis.

A 14-day old (39w0d gestational age) boy was initially seen in pediatric urology clinic for circumcision evaluation. His genital examination was unremarkable, and it was requested that he return for repeat examination and pre-operative assessment when he was 6 months of age, at which time he would be cleared to undergo general anesthesia for elective surgery, per our institutional guidelines. At his follow up visit, his right testicle was found to be small and firm with effacement of the epididymis posteriorly, initially suspicious for remote perinatal torsion. An ultrasound was ordered which showed a comparatively smaller right testicle (11 × 9 × 8 mm compared to 16 × 10 × 9 mm on the left) with adequate blood flow on doppler, and a 1.4 cm heterogeneous lesion with internal calcifications and hypoechoic rim in the right inguinal region (Fig. 1). The initial radiologic interpretation of this mass was suspicion for pilomatrixoma. Surgical exploration at the time of circumcision was offered and agreed to by the parents. The testicle was delivered through a high right hemi-scrotal incision. The testicle appeared grossly normal but there was an irregular mas inferiorly at the location of the gubernaculum. The tunica vaginalis was opened and further inspection of the testicle and epididymis did not reveal any gross abnormalities. The mass in question was clearly separate from the testicle and extra-vaginal. The overlying tissue was dissected away revealing a greenish brown irregular mass suspicious for meconium fecalith (Fig. 2). An intra-operative specimen was sent for frozen section but was ultimately inconclusive. A standard orchidopexy was then performed and the procedure concluded. Final pathology on the surgical specimen was consistent with a meconium fecalith. The patient was seen at his 6-week postoperative visit and was doing well.

## Discussion

Meconium periorchitis is often a benign finding but is sometimes associated with scrotal and testicular abnormalities such as testicular torsion, scrotoschisis, and transverse testicular ectopia [4]. Although meconium is essentially sterile due to the immature fetal gastrointestinal tract microbiome [5], there is still a significant amount of inflammation when in contact with surrounding tissues resulting in meconium peritonitis. The clinical presentation of meconium peritonitis exists on a spectrum, and management options range from conservative management in asymptomatic patients to urgent surgical intervention in those who are unstable. Given the patent internal inguinal ring in infant males, there is a direct route for communication of meconium with the scrotal contents. The classic presentation of meconium periorchitis is a soft hydrocele at birth which later results in a palpable scrotal mass as the meconium calcifies. Most cases do not require surgical intervention and result in spontaneous resolution of the calcified meconium over a period of several years [6, 7]. Regarding future prognosis, it is important to note that there is a paucity of long-term follow-up data on testicular function. Because imaging findings of a calcified scrotal mass are not specific and may be indicative of something more concerning (e.g. testicular germ cell tumor), many of these children often undergo surgical exploration. A review of 31 cases of prenatal detection of meconium periorchitis found that surgical intervention was pursued in 80.6% of cases, resulting in orchiectomy in 25% of cases, and excision of the mass in 65% of cases. Only two of the patients in this series underwent intra-operative biopsy revealing calcified meconium and, in both cases, the mass was left in-situ [8].

Khoury *et al.* have postulated three distinct mechanisms for the development of meconium periorchitis: (i) Disruption of intestinal integrity leading to spillage of meconium into the peritoneal cavity and transit into the scrotum via a patent processus vaginalis; (ii) Adherence of spilled meconium via the above mechanism to the fetal intra-abdominal testicle early in development which is then brought into the scrotum via normal transabdominal and inguinoscrotal testicular descent; (iii) Direct spillage of meconium into the scrotum via a patent processus vaginalis from a incarcerated hernia and subsequent bowel necrosis [8]. There was no evidence of antenatal or postnatal bowel pathology in our patient as the pregnancy was uncomplicated, and his only postnatal health condition included macrocephaly which resolved without intervention.

Our case is an interesting anatomical anomaly where the meconium fecalith was located outside of the tunica vaginalis. One possibility as to how the meconium could potentially escape the tunica vaginalis and deposit in the para-testicular soft tissue is via lymphatic dissemination. Theoretically, meconium extruded from the bowel could have deposited itself onto the gubernaculum prior to the testicle's descent into the scrotum. Alternatively, lymphatic stomata lining the mesothelium of the tunica vaginalis have been shown to transport molecules across the tunical layer and is the presumed mechanism of spontaneous hydrocele resolution after obliteration of the patent processus vaginalis [9, 10]. Another potential mechanism of extra-vaginal deposition of intra-abdominal meconium relates to early inflammatory disruption of tissue barriers. Meconium exposure in the peritoneal cavity is intensely pro-inflammatory and has been shown to be associated with defects in normal anatomic development of the testicle and surrounding tissues, as described above [4].

In conclusion, meconium periorchitis is an uncommon but important condition to be considered in the evaluation of the neonate. Early recognition can prevent unwarranted surgery that may result in removal or injury to an otherwise healthy, normal functioning testicle.
